# Zieve Syndrome in a Patient With Hepatitis C

**DOI:** 10.7759/cureus.25412

**Published:** 2022-05-27

**Authors:** Asad A Haider, Joshua K Salabei, Mariam Hashmi, Zeeshan Ismail, Mingyue Zheng, Uma G Iyer

**Affiliations:** 1 Internal Medicine, University of Central Florida College of Medicine, Graduate Medical Education/Hospital Corporation of America (HCA) Florida North Florida Hospital, Gainesville, USA

**Keywords:** chronic liver disease (cld), alcohol use, hepatitis c (hcv) infection, alcohol-related liver disease, s: anemia, acute hemolytic anemia, zieve's disease, zieve syndrome

## Abstract

Zieve syndrome is a very rare syndrome that presents as a triad of hemolytic anemia, jaundice, and transient hyperlipidemia in patients with alcoholic liver disease. Herein, we present a case of a 30-year-old female with alcoholic liver disease and chronic hepatitis C. She presented with altered mental status and profound jaundice and was subsequently found to have acute hemolytic anemia due to Zieve syndrome. All other causes of hemolytic anemia were ruled out. She abstained from alcohol and received blood transfusions as needed, leading to the improvement of her anemia. This case highlights the need for more medical education about Zieve syndrome as the under-recognition of the disease can lead to unnecessary treatments. We review the existing literature to explain the epidemiology, pathogenesis, diagnosis, and treatment of Zieve syndrome. This case represents a rare presentation of Zieve syndrome in a patient with hepatitis C, and we have hypothesized a possible role of chronic hepatitis C infection in its pathophysiology.

## Introduction

Zieve syndrome is a very rare syndrome that typically presents in patients with alcoholic liver disease with a triad of hemolytic anemia, jaundice, and transient hyperlipidemia. Although thought to be due to the interruption of cholesterol metabolism in the liver causing an altered phospholipid bilayer, the exact mechanism is not definitively known [1]. Diagnosis is made by the presence of hemolytic anemia, jaundice, and transient hyperlipidemia in the setting of chronic alcohol use, although lipid levels are normal in half of all patients [2]. Treatment consists of abstinence from alcohol and blood transfusions as necessary, and most patients recover within 4-6 weeks after alcohol cessation [2,3]. While there are many causes of anemia in chronic liver disease, Zieve syndrome is distinct in that it is hemolytic. Herein, we present a case of a 30-year-old female with Zieve syndrome who has a history of alcoholic liver disease and chronic hepatitis C. This case highlights the need for more medical education about this syndrome, as under-recognition can lead to unnecessary treatments and prolonged recovery.

## Case presentation

A 30-year-old female with multiple comorbidities, including chronic liver disease due to alcohol use disorder and chronic hepatitis C, presented to our facility complaining of nausea, vomiting, and shortness of breath. She had not been seen by a medical provider for many years. She had been drinking alcohol for many years prior to presentation and had never been treated for hepatitis C. The physical exam was significant for slurred speech, severe jaundice, scleral icterus, poor oral hygiene, and a distended abdomen. The patient reported no personal or family history of autoimmune disorders or congenital hemolytic anemias. She denied taking any medications associated with drug-induced hemolytic anemia, such as sulfonamides or penicillins, or having had any recent blood product transfusions.

**Figure 1:**
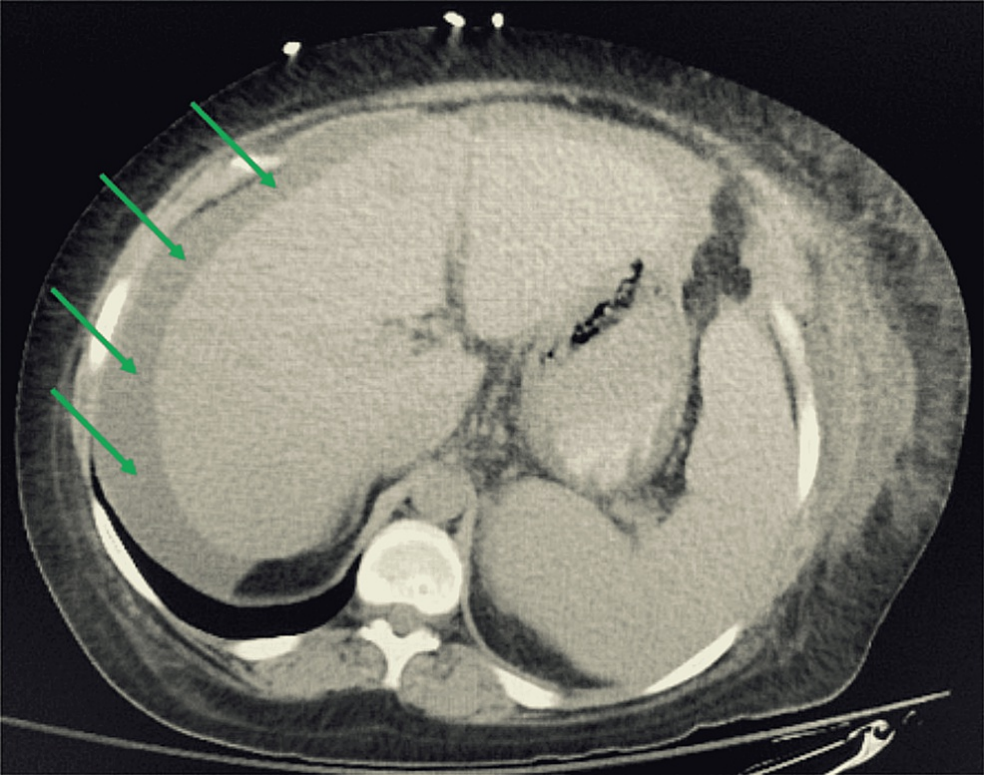
Representative CT image of the abdomen showing ascites. Green arrows indicate the location of ascites.

**Figure 2:**
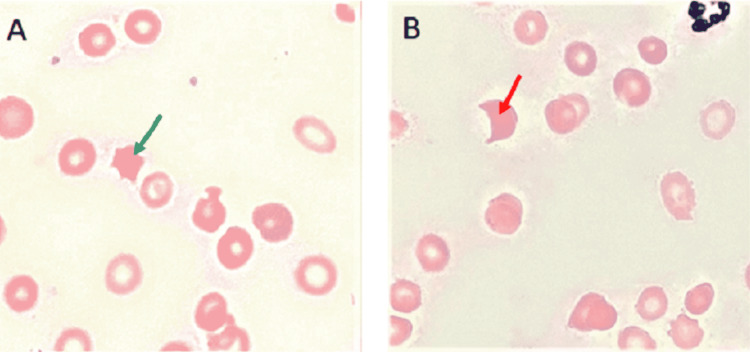
Peripheral blood smear. (A) Green arrow indicates an acanthocyte. (B) Red arrow indicates a schistocyte.

Initial lab studies were significant for a hemoglobin of 6.1 g/dL and a hematocrit of 18% (Table 1). An abdominal CT scan showed moderate ascites (Figure 1). A peripheral blood smear showed acanthocytes and schistocytes (Figure 2). Two units of packed RBCs were transfused on day 1 of admission.

**Table 1:** Pertinent laboratory data at the time of presentation. LDH: Lactate dehydrogenase; INR: International normalized ratio; PTT: Partial thromboplastin time.

Labs	Levels on admission	Normal range
WBCs	15.5	(4.5-11.0 thousand/mm^3^)
Hemoglobin	6.1	(11.2-15.7 g/dL)
Hematocrit	18.0	(34.1-44.9%)
Platelet count	185	(150-400 thousand/mm^3^)
Sodium	130	(136-145 mmol/L)
Potassium	2.0	(3.5-5.1 mmol/L)
Creatinine	1.34	(0.60-1.30 mg/dL)
Glucose	107	(74-106 mg/dL)
Calcium	7.9	(8.5-10.1 mg/dL)
Aspartate aminotransferase	105	(15-37 units/L)
Alanine aminotransferase	41	(13-56 units/L)
Alkaline phosphatase	101	(45-117 units/L)
Total bilirubin	12.1	(0.2-1.0 mg/dL)
Ammonia	102	(11-32 micromol/L)
LDH	251	(84-246 units/L)
Reticulocyte count	8.12	(0.50-1.70%)
INR	3.9	(0.8-1.1)
PTT	41.4	(25-38 seconds)
Hepatitis C antibody	Positive	

A direct Coombs test was negative, which ruled out autoimmune hemolytic anemia. Zieve syndrome was thus considered to be the cause of her hemolytic anemia. Of note, the hyperlipidemia seen in Zieve syndrome is transient and can occur months before its diagnosis; therefore, the lipid panel can be normal at the time of diagnosis, as seen in our patient (Table 2).

**Table 2:** Lipid panel values. LDL: Low-density lipoprotein; HDL: High-density lipoprotein.

Labs	Level	Normal range
Triglycerides	53	(0-149 mg/dL)
LDL cholesterol	10	(100-159 mg/dL)
HDL cholesterol	29	(40-60 mg/dL)

**Figure 3:**
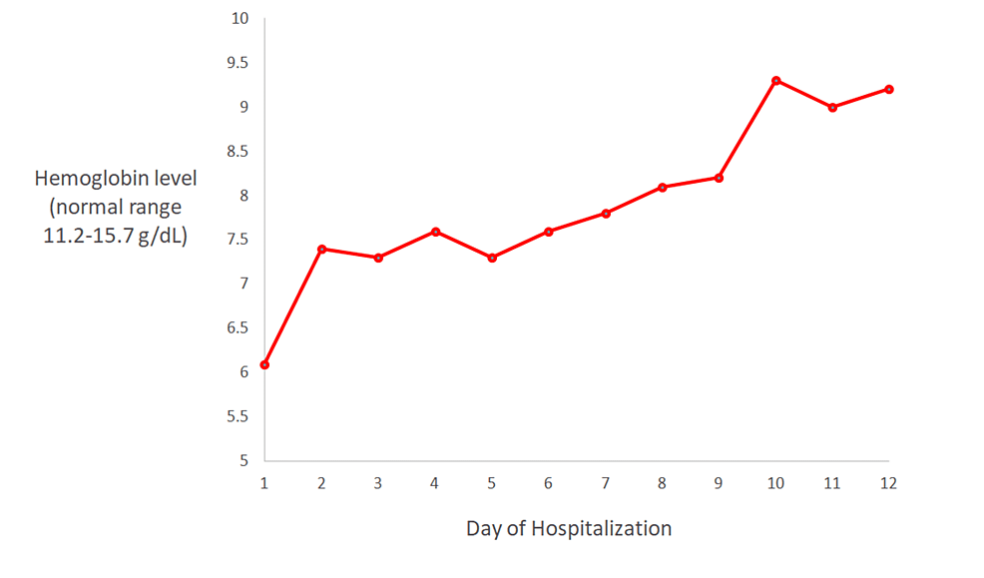
Hemoglobin values throughout hospitalization.

This patient’s diagnosis of Zieve syndrome was based on the features of alcoholic liver disease, hemolytic anemia, severe jaundice, peripheral blood smear findings of acanthocytes and schistocytes, and no other identifiable cause of hemolytic anemia. The patient was advised to abstain from alcohol. Other than the two units of packed RBCs that the patient received on day 1 of admission, she did not receive further blood product transfusions. Her anemia gradually improved, and she was discharged on day 12 of admission (Figure 3). Shortly after discharge, she started treatment for hepatitis C with sofosbuvir-velpatasvir, which led to further improvements in her hemoglobin and total bilirubin levels one month after discharge. Her hemoglobin level (normal range: 11.2-15.7 g/dL) improved from 9.2 g/dL at the time of discharge to 11.2 g/dL at outpatient follow-up one month later. Her total bilirubin level (normal range: 0.2-1.0 mg/dL) improved from 7.3 mg/dL at the time of discharge to 4.1 mg/dL at outpatient follow-up one month later.

## Discussion

Zieve syndrome, a rare syndrome that consists of a triad of hemolytic anemia, jaundice, and transient hyperlipidemia, was first described in 1958 by Dr. Zieve [4]. The jaundice is profound due to the build-up of bilirubin, both directly from hepatocyte injury and indirectly from hemolysis [2]. Zieve syndrome is frequently under-reported and misdiagnosed. About 200 cases have been described in the literature [5]. A literature search using the PubMed database uncovered 123 publications related to Zieve syndrome, with reported periods of 1958 to 2022. 

The exact mechanism of Zieve syndrome is not definitively known. The most well-supported hypothesis asserts that dysregulation of hepatic lipases due to a damaged liver causes a massive mobilization of fat and ensuing hyperlipidemia [2]. Hyperlipidemia causes direct injury to hepatocytes and results in high levels of lysolecithin and lysocephalin, which can trigger the process of hemolysis [1]. Another proposed hypothesis for the pathogenesis of Zieve syndrome asserts that alcohol-induced vitamin E deficiency leads to a reduction in erythrocyte glutathione and polyunsaturated fatty acids. This can cause plasma membrane instability in RBCs and eventually hemolysis [6]. Other suggested mechanisms of Zieve syndrome include pyruvate kinase instability or inhibition of RBC enzymes by acetaldehyde, a reactive metabolite of ethanol [7].

Our patient’s diagnosis of Zieve syndrome was established based on the presence of profound jaundice, hemolytic anemia, an alcohol use disorder, and characteristic peripheral blood smear findings of acanthocytes and schistocytes [8]. Although transient hyperlipidemia is considered a classic finding in Zieve syndrome, lipid levels can be normal on presentation in up to half of patients, as detection and diagnosis can occur months after the initial massive mobilization of fat [8]. Our patient had little medical care prior to this hospitalization. She likely had this event of fat mobilization occur many months prior to the presentation, which explains her normal lipid levels. After abstinence from alcohol, she gradually recovered. These findings support the diagnosis of Zieve syndrome.

The treatment of Zieve syndrome consists of abstinence from alcohol, blood transfusions as needed, and general supportive treatment [2]. Alcohol cessation causes lipases to become more readily activated, causing lipids to be deposited in adipose tissue. This lowers the amount of lipids in the plasma, thus reducing hemolysis and resultant jaundice. The prognosis of this disease is very good, and the vast majority of patients recover within 4-6 weeks of beginning treatment [2]. In one case, a patient with triglyceride levels >4,000 mg/dL was treated with plasmapheresis [9]. Patients with Zieve syndrome who have a history of pancreatitis or intracerebral hemorrhage should be evaluated for plasmapheresis, as elevated lipid levels are known to result in worse outcomes in patients with pancreatitis or intracerebral hemorrhage [3,10]. Importantly, corticosteroids have not been shown to be beneficial in treating Zieve syndrome and should be avoided [11]. Our patient recovered in the expected time frame after beginning treatment.

It is very important to recognize Zieve syndrome in order to prevent complications from misdiagnosis and inappropriate treatment. In some cases, patients have been started empirically on corticosteroids for presumed autoimmune hemolytic anemia, only to be discontinued later when a direct Coombs test is negative. Using corticosteroids in severely ill patients has been linked to an increased incidence of hospital-acquired infections, so it is essential to consider Zieve syndrome in the differential diagnosis in the setting of hemolytic anemia in a patient with alcohol use disorder [12]. In addition, patients with Zieve syndrome commonly present in the context of alcoholic hepatitis. The Maddrey discriminant function for alcoholic hepatitis is a commonly used scoring system that incorporates total bilirubin and prothrombin time to calculate disease severity and prognosis [13,14]. A score above 32 suggests that the patient might benefit from corticosteroid treatment. However, bilirubin levels are expected to be significantly elevated in Zieve syndrome. Failure to consider the effect of Zieve syndrome when using the Maddrey discriminant function can lead to a falsely elevated score and unnecessary corticosteroid use. Improved recognition of Zieve syndrome can improve patient outcomes and prevent unnecessary complications.

Finally, this is the first reported case, to date, of a patient with Zieve syndrome who also has hepatitis C. As Zieve syndrome is caused by liver damage, it is reasonable to hypothesize whether the interaction of hepatitis C virus with the liver contributed to the pathogenesis of Zieve syndrome in this patient. Hepatitis C is known to disrupt lipoprotein lipase. Since Zieve syndrome is thought to be caused by dysregulation of hepatic enzymes, this might be a mechanism by which hepatitis C could contribute to Zieve syndrome [15]. This patient’s Zieve syndrome improved one month after hospital discharge. At that time, she had been undergoing treatment for hepatitis C. Because she had also been abstaining from drinking alcohol during this time, it is difficult to separate the role of hepatitis C treatment in the resolution of Zieve syndrome, if at all there is one. Further clinical and molecular studies need to be done to elucidate whether hepatitis C plays a role in Zieve syndrome.

## Conclusions

Here, we have presented a case of a patient with Zieve syndrome, a very rare cause of hemolytic anemia. We have also reviewed the existing literature to explain the epidemiology, pathogenesis, diagnosis, and treatment of Zieve syndrome. As the incidence of chronic liver disease and alcohol use disorder increases, Zieve syndrome will become more common. Therefore, it is essential to improve awareness of this syndrome. As research and education about Zieve syndrome increase, we anticipate seeing an improvement in patient recovery times and fewer delays in diagnosis.
